# Host Shifts from Lamiales to Brassicaceae in the Sawfly Genus *Athalia*

**DOI:** 10.1371/journal.pone.0033649

**Published:** 2012-04-02

**Authors:** Sebastian E. W. Opitz, Jean-Luc Boevé, Zoltán Tamás Nagy, Gontran Sonet, Frank Koch, Caroline Müller

**Affiliations:** 1 Department of Chemical Ecology, Bielefeld University, Bielefeld, Germany; 2 IRSNB-KBIN, Royal Belgian Institute of Natural Sciences, Bruxelles, Belgium; 3 Museum für Naturkunde, Leibniz-Institut für Evolutions- und Biodiversitätsforschung an der Humboldt-Universität zu Berlin, Berlin, Germany; University of Arkanas, United States of America

## Abstract

Plant chemistry can be a key driver of host shifts in herbivores. Several species in the sawfly genus *Athalia* are important economic pests on Brassicaceae, whereas other *Athalia* species are specialized on Lamiales. These host plants have glucosides in common, which are sequestered by larvae. To disentangle the possible direction of host shifts in this genus, we examined the sequestration specificity and feeding deterrence of iridoid glucosides (IGs) and glucosinolates (GSs) in larvae of five species which either naturally sequester IGs from their hosts within the Plantaginaceae (Lamiales) or GSs from Brassicaceae, respectively. Furthermore, adults were tested for feeding stimulation by a *neo*-clerodane diterpenoid which occurs in Lamiales. Larvae of the Plantaginaceae-feeders did not sequester artificially administered *p*-hydroxybenzylGS and were more deterred by GSs than Brassicaceae-feeders were by IGs. In contrast, larvae of Brassicaceae-feeders were able to sequester artificially administered catalpol (IG), which points to an ancestral association with Lamiales. In line with this finding, adults of all tested species were stimulated by the *neo*-clerodane diterpenoid. Finally, in a phylogenetic tree inferred from genetic marker sequences of 21 *Athalia* species, the sister species of all remaining 20 *Athalia* species also turned out to be a Lamiales-feeder. Fundamental physiological pre-adaptations, such as the establishment of a glucoside transporter, and mechanisms to circumvent activation of glucosides by glucosidases are therefore necessary prerequisites for successful host shifts between Lamiales and Brassicaceae.

## Introduction

Most phytophagous insects are diet specialists and depend on a limited number of plant species that can serve as suitable hosts [1], [2]. Feeding specialization is driven mainly by the specific composition of secondary plant metabolites [3], and herbivores are adapted to these metabolites by various behavioral and/or physiological means such as sequestration, i.e., the uptake, concentration and storage of these compounds [4].

Different scenarios have been discussed concerning how herbivorous insects are able to add new host plant species to their diet in the course of evolution. Ehrlich and Raven [5] proposed the escape and radiation hypothesis to explain the evolution of plant herbivore interactions. They suggested that plants escape herbivory by evolving new defense chemicals followed by radiation of the plant species. Herbivores can shift to these plants if they can overcome plant defenses and then radiate as well [5], [6]. Phytochemical similarity between the ancestral and new host plants is probably necessary to facilitate such shifts by the herbivores [7]–[9], whereas phylogenetic similarity between plant species is regarded as less important [10], [11].

The oscillation hypothesis postulates that host shifts do not happen abruptly. Instead, before insect species entirely shift to a new host, it is expected that over rather long time periods host plant ranges are extended to include both the old and new plant species [12], [13]. It may also be expected that when speciation occurs involving a host shift, the descendant species on the new host maintains some ability to deal with secondary metabolites of the ancestral host. In contrast, the descendant species remaining on the ancestral host may have less ability to handle specific metabolites of the new host because it never experienced it. Apart from plant chemistry, ecological factors such as a shared habitat may also explain why herbivores may switch to new host species [14], [15]. Overall, host shifts within a plant genus or family are more likely [16]–[18], and there is evidence that only a small proportion of insect speciation events include shifts towards a different plant family [19], [20]. Host shifts to distant families occur more likely in those insect lineages that include polyphagous species and that are geographically widespread [21], [22].

The tenthredinid sawfly genus *Athalia* comprises about 70 species and subspecies that are oligophagous but are specialized on different plant families, namely either on species in Plantaginaceae, Lamiaceae (both Lamiales), Brassicaceae and Tropaeolaceae (Brassicales), or Crassulaceae (Saxifragales) [23], [24] (Table 1). On cultivated crucifers, sawfly larvae can be notorious pests [23]. Their host specialization patterns on distantly related plant families and their economic significance make them an intriguing model to study the evolution of host plant shifts in this genus.

**Table 1:** Species of *Athalia* included in the present study, with their host plant associations and geographic distribution.

Species	Host plant families of larvae	Refererences for host association	Geographic distribution
*A. ancilla* Serville, 1823	Brassicaceae	[24]	West Palaearctic
*A. bicolor* Serville, 1823		b	West Palaearctic
*A. circularis* (Klug, 1815)	(Asteraceae, Brassicaceae, Lamiaceae), Plantaginaceae^1^	[24], a	Palaearctic
*A. cordata* Serville, 1823	Lamiaceae, Plantaginaceae	[24], a	West Palaearctic
*A. cornubiae* Benson, 1931	Crassulaceae	[24]	Palaearctic
*A. excisa* Koch, 2006	Brassicaceae	[65], a	Afrotropic
*A. flavobasalis* Koch, 2007	Brassicaceae	a	Afrotropic
*A. guillarmodi* Benson, 1956	Brassicaceae	[23], [65], a	Afrotropic
*A. himantopus* Klug, 1834	Brassicaceae	[23], [66], a	Afrotropic
*A. incomta* Konow, 1908	Lamiales	a	Afrotropic
*A. liberta* (Klug, 1815)	Brassicaceae	[24], a	Palaearctic
*A. lugens* (Klug, 1815)	Brassicaceae	[67], a	Palaearctic
*A. marginipennis* Enderlein, 1920	Brassicaceae	[66] a	Afrotropic
*A. obsoleta* Benson, 1962	Brassicaceae	a	Afrotropic
*A. rosae rosae* (Linné, 1758)	Brassicaceae, Tropaeolaceae^2^	[24], a	Palaearctic, Oriental
*A. rosae ruficornis* Jakovlev, 1888	Brassicaceae	[24], a	East Palaearctic, Oriental
*A. scioensis* Gribodo, 1879		b	Afrotropic
*A. scutellariae* Cameron, 1880	Lamiaceae	[24]	Palaearctic
*A. ustipennis* Mocsáry, 1909	Brassicaceae	a	Afrotropic
*A. vollenhoveni* Gribodo, 1879	Brassicaceae	[23]	West Palaearctic, Afrotropic

1 Our observations with various field collected and reared animals revealed only Plantaginaceae as host plants for successful development; ^2^our observations revealed only Brassicaceae as host plants for successful reproduction; a. our results from chemical analyses; b. host plants unknown, in chemical analyses neither GSs nor aucubin, catalpol or verbascoside could be detected. Geographic distribution according to [30].

The larvae of several Plantaginaceae-feeders, including *A. cordata* and *A. circularis*, sequester iridoid glucosides (IGs) such as catalpol and aucubin in their hemolymph [25]. Conversely, larvae specialized on Brassicaceae, e.g., *A. rosae rosae*, *A. liberta* and *A. lugens*, were found to sequester glucosinolates (GSs) [25]. Sequestered IGs and GSs are used for the larvae’s own defense against invertebrate predators [25], [26] while adult *Athalia* feed mainly on nectar. However, adults of the Japanese subspecies *A. rosae ruficornis* have been found to acquire *neo*-clerodane diterpenoids from Lamiaceae and Verbenaceae (Lamiales) by feeding on the leaf surface, and even use these compounds for defense and in sexual communication [27], [28]. These diterpenoids occur in plant species of the Lamiales, but not in Brassicales [29].

To shed light on the evolutionary trajectory of host shifts within the genus *Athalia*, we took an integrated approach by addressing whether the larvae of five European species specialized on Plantaginaceae or Brassicaceae are able to sequester both IGs and GSs, and whether they are stimulated or deterred by these glucosides. Furthermore, adults were tested for their stimulation to a *neo*-clerodane diterpenoid. Finally, a molecular phylogenetic tree was reconstructed, including European as well as African and one Japanese (sub-)species, as the genus is distributed in the Palaearctic and African regions [23], [30]. We discuss the significance of our findings in the framework of existing theories of the evolution of plant-insect associations, and suggest that these theories are not mutually exclusive.

## Materials and Methods

### Insect and Plant Rearing

*Athalia circularis*, *A. cordata*, *A. rosae rosae*, *A. liberta*, and *A. lugens* were collected around Würzburg and Bielefeld, Germany, in 2010 and kept in cages in a greenhouse (20°C, photoperiod of L16:D8). Host plants were grown from seeds in the greenhouse. Seeds of *Plantago lanceolata* L. (Plantaginaceae) were obtained from Rühlemann′s (Horstedt, Germany) and seeds of *Veronica beccabunga* L. (Plantaginaceae) and *Alliaria petiolata* (M. Bieb.) Cavara & Grande (Brassicaceae) from the Botanical Garden of Berlin, Germany. *Veronica beccabunga* was sown once and subsequently propagated by cuttings. Seeds of *Sinapis alba* L. cv. Silenda (Brassicaceae), *Nasturtium officinale* (R. Br.), and *Brassica rapa* L. (var. *pekinensis*) (Brassicaceae) were obtained from Kiepenkerl (Norken, Germany). Within the specialists on Plantaginaceae, A. *cordata* was reared on *P. lanceolata* and *A. circularis* on *V. beccabunga*. Within the specialists on Brassicaceae, *A. rosae rosae* was kept on *S. alba*, *A. liberta* on first-year *A. petiolata* plants, and *A. lugens* on *N. officinale*. Adults were provided with a mixture of honey and water (1∶50). Sawflies of *A. circularis, A. cordata* and *A. rosae rosae* could be reared through several generations, whereas only few females of *A. liberta* and *A. lugens* were found in the field. Females of the latter two species produced only few offspring and could not be further reared. Therefore, the number of individuals of these species available for bioassays was limited. All species were used for bioassays in the first to third generation after collection in the field. No specific permits were required for our described bioassays.

### Testing for Sequestration Abilities of Non-Host Glucosides

Potential sequestration abilities of non-host glucosides were tested with these five *Athalia* species. Non-host glucosides were dissolved in 90% methanol, applied on larval host plant leaf discs (diameter: 10 mm) in a naturally occurring concentration (7 µmol/g FW, equals between 250 and 350 nmol per leaf disc) and solvents were allowed to dry. Painted leaf discs were offered individually to single last feeding instar larvae in glass Petri dishes (5 cm diameter). *p*-HydroxybenzylGS (sinalbin; Phytoplan) was applied on *P. lanceolata* and *V. beccabunga* leaf discs to test the sequestration ability of a GS as non-host glucoside by *A. cordata* and *A. circularis*. Catalpol (Phytoplan) was applied on *S. alba, A. petiolata* and *N. officinale* leaf discs to test sequestration of an IG as non-host glucoside by *A. rosae rosae, A. liberta*, and *A. lugens*, respectively. Larvae were starved 2 to 12 h prior to the feeding bioassays, depending on the size and feeding activity of the species. After 5 h of feeding, hemolymph was taken from each individual by piercing the integument with a needle and collecting the exuding hemolymph droplet. Additionally, feces and remaining leaf parts were collected from each individual in Eppendorf caps. Control leaf discs were sampled directly after application of the glucoside and used to calculate maximum available glucoside values. All samples were frozen at –80°C for later analyses of glucoside concentrations.

### Testing for Feeding Deterrence of Non-Host Glucosides

The deterrent activity of non-host glucosides was investigated for one Plantaginaceae- and one Brassicaceae-feeding species in paired choice feeding assays. Individual last feeding instar larvae of *A. cordata* (Plantaginaceae-feeder) were offered one test leaf disc (12 mm diameter) of *P. lanceolata* treated with *p*-hydroxybenzylGS (dissolved in 90% methanol) and a control disc treated only with solvent in a Petri dish. Larvae of *A. rosae rosae* (Brassicaceae-feeder) were offered a test leaf disc of *S. alba* treated with the IG catalpol and a control disc treated with solvent. Non-host glucosides were applied in approximately the same glucoside concentrations (360 nmol per leaf disc) as used for the sequestration assays (see above). After 3 h, feeding amounts on both discs were measured in mm^2^ with mm^2^-paper and data compared by Wilcoxon matched-pairs signed-rank tests to evaluate possible deterrent effects of the non-host glucosides on each species. For comparison of the deterrent effectiveness of non-host glucosides on *A. cordata* and *A. rosae rosae*, preference indices (PI) were calculated [PI = (amount consumed on test – amount consumed on control) / total amount consumed] and compared with a Mann-Whitney *U*-test.

### Analyses of Glucoside Concentrations in Larvae

GS and IG concentrations of larval hemolymph, feces and leaf samples were determined by HPLC-DAD and GC-MS, respectively, according to [25], using 2-propenylGS (Phytoplan) or phenyl-β-D-glucopyranoside (Sigma-Aldrich) as internal standards.

### Testing for Feeding Stimulation by Clerodendrin B

To test whether feeding of adult *Athalia* species was stimulated by a representative *neo-*clerodane diterpenoid, females were offered clerodendrin B in paired choice assays. Clerodendrin B (1 µg in 10 µl ethyl acetate, kindly purified and provided by R. Nishida; test) or 10 µl ethyl acetate only (control) were applied on two green paper napkin squares (64 mm^2^) and placed in a glass Petri dish. The feeding durations on test and control squares of individually tested 3 to 6 d old naïve females of *A. cordata*, *A. circularis, A. rosae rosae*, *A. liberta*, and *A. lugens* were recorded during 5 min in the paired choice assays. The feeding stimulation reaction was scored as positive when individuals fed continuously on the test paper for more than 10 s [31]. A Kruskal-Wallis analysis of ranks followed by a multiple comparison test was used to compare the feeding times of *Athalia* species on the filter papers treated with clerodendrin B.

### Larval Host Associations Deduced from Chemical Analyses of Preserved Adult Specimens

Glucosides sequestered by *Athalia* larvae are transferred to the adult stage [32], and adults are more easily collected in the field and taxonomically much better studied than larvae. Therefore, preserved adult specimens of several species collected in Europe, Africa and Japan were analyzed for the presence of glucosides to determine or verify the host plant associations of these specimens (Table 1). Sampled adults were either preserved by drying or in ethanol for up to 15 years. For the samples collected in Ethiopia (during October 2010, Tab. 1, S1) a necessary ‘Material Transfer Agreement’ and a ‘Material Export Permit’ were obtained from the Institute of Biodiversity Conservation (Addis Ababa). Some of the Afrotropical *Athalia* species were collected during the project “The sawfly diversity of the Afromontane Region of South Africa”. This research was supported and permitted by Mpumalanga Tourism and Parks Agency, Limpopo Provincial Government, Department of Economic Development, Environment and Tourism, as well as Ezemvelo KZN Wildlife, Conservation, Partnerships and Ecotourism. For all other specimens, no specific permits were required for the sampling activities since the collection sites were not privately owned or protected in any way. None of these studies involved endangered or protected species. In many cases only one individual per species was available. Abdomens of each individual were ground, extracted in methanol (HPLC-grade, Merck), and analyzed on a LC-18 column (Grom-Sil, 120 ODS-4 HE, 3 µm, 150×2 mm, Alltech Grom) by UHPLC-TOF-MS (1290 series UHPLC, 6210 series Time-of-Flight, Agilent Technologies). Samples were eluted at a gradient from 0.1% formic acid (Merck; solvent A) to acetonitrile (Fisher Scientific; with 0.1% formic acid; solvent B) and a flow of 0.6 ml/min at a column temperature of 35°C. For GS detection, samples were measured in negative ionization mode with an ESI source (Dual-ESI, drying gas 12 I/min, fragmentor 140 V, gas temperature 350°C, nebulizer 55 psig, Oct 1 RF Vpp 250 V, skimmer 60 V, vaporizer/sheath gas temperature 375°C, VCap 3491 V). For IG and verbascoside analysis, all samples were measured again in positive ionization mode with higher fragmentor voltage (170 V). Reference masses were used for internal mass calibration during the runs, introduced by a second sprayer in the source. The corresponding glucosides were identified by their exact masses and calculated sum formulae, their UV-spectra, and by comparison with authentic standards. For IG detection, some of the samples were additionally analyzed by GC-MS as described in [25].

### Genetic Analysis of *Athalia* and Host Plant Relationships

Legs of pinned or ethanol-preserved adults as well as hind parts of larvae were used for DNA extraction (for collection data see Table S1). Total genomic DNA was extracted using the NucleoSpin Tissue kit (Macherey-Nagel) following the manufacturer’s instructions. Two genetic markers were selected: a fragment of the mitochondrial cytochrome oxidase I (COI; aligned length: 801 bp) gene was amplified and sequenced with the primers sym-C1-J-1718 [33] and A2590 [34]. For three specimens of *A. lugens*, only shorter DNA fragments (418 bp overlap with the longer COI sequences) could be amplified with primers LCO1490 and HCO2198 [35]. A fragment of the nuclear 28S rRNA (28S, aligned length: 583 bp) gene was amplified and sequenced with D2F and D2R [36]. PCR products were purified using NucleoFast 96 PCR plates (Macherey-Nagel) and directly sequenced on an ABI 3130xl automated capillary sequencer (Life Technologies) using BigDye v1.1 chemistry. DNA sequences were assembled and checked using SeqScape v2.5 (Applied Biosystems), 28S sequences were aligned with the MAFFT 6 web server [37], [38]. PAUP* v4b10 [39] was used to calculate maximum parsimony trees. Node supports were assessed by non-parametric bootstrapping (2,000 bootstrap replicates). For a Bayesian inference of phylogeny, MrBayes v3.1.2 [40] was used. The whole data set was partitioned according codon positions and genetic markers, so a total of four data partitions were used, i.e. three partitions for the COI data, and one partition for the 28S data. Appropriate nucleotide substitution models for each partition were selected by jModeltest 0.1.1 [41] using the Bayesian Information Criterion (BIC). Two parallel runs, each including four Markov chains, were run for 5 million generations, with every 1000th tree sampled. Convergence was checked by Tracer v1.5 [42], and the first 10% of the trees were discarded as burn-in. All trees were rooted with *Arge ustulata, Giladeus tuxius* and *Gilpinia hercyniae.*

In order to reveal ancestral associations between *Athalia* larvae and their host plants, a reconstruction of ancestral character states (host plant) was performed for each internal node of the produced phylogenetic tree using the most parsimonious character reconstruction (MPR) as described in [43] and modified by [44], and maximum likelihood (ML) with equal rates and symmetric models in R (version 2.13.2) with the package ape 2.8 [45]. GenBank accession numbers can be found in Table S1.

## Results

### Sequestration of Non-Host Glucosides

**Figure 1:**
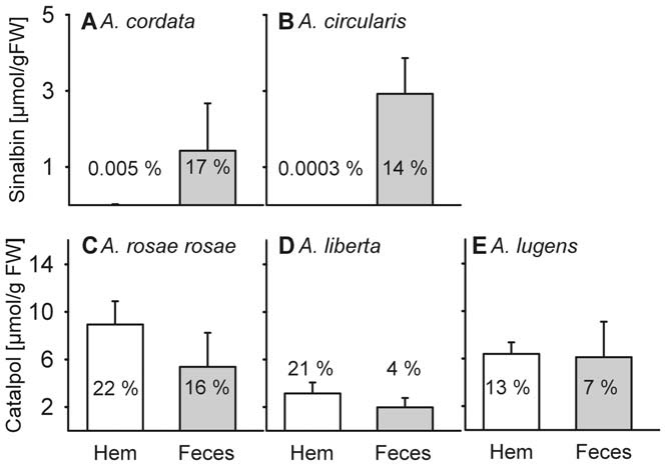
Non-host glucoside concentrations (mean±SD) in the larval hemolymph (Hem, *white bars*) and in the feces (*grey bars*) of five *Athalia* species (last feeding instar, n = 6) after 5 **h of feeding: (A)** ***A. cordata*** **fed on** ***Plantago lanceolata*** **and (B)** ***A.*** ** ***circularis*** **fed on** ***Veronica beccabunga*** **, (A, B) treated with** ***p*** **-hydroxybenzylGS; (C)** ***A. rosae rosae*** **fed on** ***Sinapis alba*** **, (D)** ***A. liberta*** **fed on** ***Alliaria petiolata*** **, and (E)** ***A. lugens*** **fed on** ***Nasturtium officinale*** **, (C, E) treated with catalpol (IG).** The relative amounts of non-host glucoside present in either hemolymph or feces compared to what was theoretically ingested is given as an average percentage.

When the Plantaginaceae-feeding species *A. cordata* and *A. circularis* fed on host leaves treated with *p*-hydroxybenzylGS, only minute traces of this GS (ca. 0.01 % of theoretically ingested amounts) were detected in the hemolymph of some individuals (*A. circularis*: 1 of 6; *A. cordata*: 3 of 6). Instead, 20–25% of the ingested GS were excreted with the feces after 5 h (Fig. 1A–B). In contrast, the Brassicaceae-specialists *A. rosae rosae, A. liberta*, and *A. lugens* were able to sequester the non-host IG catalpol in their hemolymph when fed with IG-treated host plant leaves (Fig. 1C–E). The total concentration of sequestered catalpol in the hemolymph of *A. rosae rosae* accounted for about 22 % of theoretically ingested catalpol. Total IG concentrations in the hemolymph of *A. rosae rosae* were higher than in *A. liberta* and *A. lugens*, but the sequestration efficiencies of theoretically ingested catalpol were comparable (Fig. 1). Differences in IG concentrations found in the larvae may be due to lower feeding activities by the latter two species, and to interspecific variation in body masses (mean fresh mass±1 SD of larvae was 59.5±6.4 mg in *A. rosae rosae* compared to 27.3±5.7 mg in *A. liberta*; n = 6 per species). The experiment was replicated several times (between 6 and 18 individuals per species, depending on availability) with the same qualitative outcome regarding the sequestration pattern. However, only the most representative data set is shown in Fig. 1. Here, larvae of all species fed comparable amounts of leaf tissue on homogenous leaf material quality, enabling quantitative analyses.

### Feeding Deterrence of Non-Host Glucosides

Both *Athalia* species significantly preferred to feed on control leaves compared to leaf discs treated with a non-host glucoside (Table 2). However, *A. cordata* larvae were significantly more deterred by their non-host glucoside *p*-hydroxybenzylGS (mean preference index, PI, ±1 SD: –0.76±0.41, n = 12) than *A. rosae rosae* larvae by catalpol (PI: –0.33±0.30, n = 17; Mann-Whitney *U*-Test, W = 157, P = 0.014).

**Table 2:** Feeding amounts [mm^2^] of last instar larvae of *Athalia rosae rosae* and *A. cordata* on host plant leaf discs (113 mm^2^) treated with non-host glucosides dissolved in 90% methanol (7 µmol/g FW; test disc) or 90% methanol (control disc) in paired choice assays for 3 h.

Insect species – test plant species	n	Feeding amount on test disc (mean±SD)	Feeding amount on control disc (mean±SD)	P-value (Wilcoxon matched-pairs signed rank test)
*A. cordata* – *Plantago lanceolata* (+ GS)	12	3.1±7.9	30.1±20.0	0.004
*A. rosae rosae* – *Sinapis alba* (+ IG)	17	38.7±19.4	77.2±24.3	<0.001

Non-host glucosides were *p*-hydroxybenzylGS (GS: glucosinolate) and catalpol (IG: iridoid glucoside).

### Feeding Stimulation by Clerodendrin B

Most naïve adult females of all investigated *Athalia* species were stimulated to feed on clerodendrin B-treated paper discs, whereas none fed on solvent-treated control discs in paired choice assays (Table 3). Overall, no significant differences were detected in feeding durations on the diterpenoid among the species (Kruskal-Wallis analysis of ranks, P>0.05).

**Table 3:** Feeding time [seconds] within 5 min of naïve female adults of five *Athalia* species on green paper squares (64 mm^2^) treated with 1 µg of clerodendrin B solved in 10 µl ethyl acetate (test square) or treated with the solvent only (control square).

	Number of individuals tested	Number of stimulated individuals	Feeding time on test square, (mean±SD)	Feeding time on control square
*A. cordata*	17	10	77.6±87.5	0
*A. circularis*	13	11	131.4±91.4	0
*A. rosae rosae*	15	14	152.2±74.2	0
*A. liberta*	4	4	162.3±84.1	0
*A. lugens*	2	2	229.0	0

Stimulation was recorded when individuals fed continuously for >10 seconds on one square. Number of replicates differs due to availability of species.

### Larval Host Associations of Preserved Adult Specimens

Various GSs (e.g., methylGS, 2-propenylGS, 6-methylsulfinylhexylGS) were found in *A. excisa*, *A. flavobasalis, A. guillarmodi*, *A. himantopus*, *A. marginipennis*, *A. obsoleta*, *A. rosae ruficornis*, and *A. ustipennis*, revealing a likely larval association with Brassicales (Table 1). In *A. incomta*, high concentrations of the phenylpropanoid glucoside verbascoside as well as low concentrations of catalpol were found which points to an association of this species with Lamiales. In *A. bicolor* and *A. scioensis*, neither GSs nor IGs nor verbascoside were detected. For *A. circularis*, use of several other plant families has been reported [24]. However, our own tests revealed that larvae of *A. circularis* collected in different years in the field and reared in the lab only feed and develop successfully on Plantaginaceae.

### Phylogeny of *Athalia* and Host Plant Relationships

**Figure 2:**
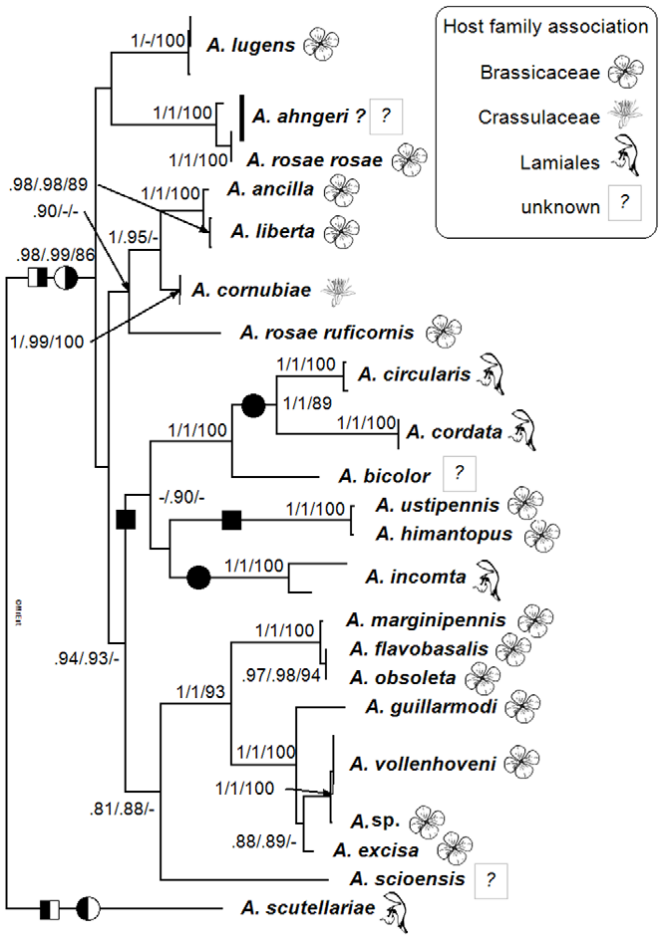
Bayesian tree of the concatenated data set showing phylogenetic relationships among 21 *Athalia* species from Europe, Africa and Japan. Numbers represent posterior probabilities obtained for the complete data set/posterior probabilities obtained after excluding the shorter COI sequences/bootstrap values of the maximum parsimony analysis. Values below .80/.80/80 are not shown. Outgroup taxa are not shown. The identity of *A. ahngeri* is uncertain. Flower symbols indicate host family associations according to Table 1. Note that we only include Lamiales as hosts for *A. circularis*, because they did not fully develop on any other host plant family except Plantaginaceae in our assays. Similarly, only Brassicaceae were included for *A. rosae rosae*. Potential host shifts hypothesized by the MPR (circles) and ML (squares) analyses are represented in the case of an ancestral association with Brassicaceae (black left-side) or Lamiales (black right-side). The figure was edited with MrEnt v.2.2^TM^.

In the phylogenetic tree, *A. scutellariae* was revealed as the sister group of all remaining ingroup species used in this study (Fig. 2). This species feeds on Lamiaceae [24]. The phylogeny and character reconstruction analyses indicated at least three host shifts in either direction, from the Lamiales to the Brassicaceae or the opposite (Fig. 2).

## Discussion

Sawfly larvae of the genus *Athalia* are specialized on different plant families. Such host shifts across plant families are rather rare and more likely found in polyphagous species [16], [18], [22]. From the distinct sequestration abilities of plant secondary metabolites found in larvae of five *Athalia* species and on the feeding stimulation pattern in the adults, we postulate that host plant shifts have occurred within the genus *Athalia* from Lamiales to Brassicaceae. Regarding our more closely studied *Athalia* species, their host plant families Plantaginaceae and Brassicaceae are only distantly related [46] and produce different secondary plant metabolite classes, IGs and GSs, which are both glucosides.

In our manipulation experiments, the Plantaginaceae-specialists *A. cordata* and *A. circularis*, which are known to sequester IGs [25], could not store GSs. In contrast to the Plantaginaceae-feeders, the three Brassicaceae-feeders, which take up and store GSs readily [25], also sequestered the non-host glucoside catalpol (IG) when offered on host leaves. Catalpol was present in *A. rosae rosae* larval hemolymph after five hours of feeding in concentrations comparable to the GS-concentrations larvae sequestered from their host plant *Sinapis alba* [25]. Given the documented sequestration ability of catalpol in all five species, it is likely that the association of *Athalia* species to IG-containing plants is ancestral. Thus, the ability to handle glucosides through extended sequestration may have facilitated the host plant shift from Lamiales to Brassicaceae.

From a physiological perspective, the putative transporter(s) for glucosides across the gut membrane seem to be less selective in the Brassicaceae-feeding *Athalia* species compared to the Plantaginaceae-specialists, the latter being obviously restricted to the transport of IGs. The broader selectivity of the putative glucoside transporter in the Brassicaceae-feeders may allow occasional and recurrent utilization of the ancestral host, which may therefore provide sufficient selection pressure to maintain this trait. As a further physiological prerequisite, all *Athalia* species investigated here need to circumvent a degradation of glucosides by glucosidases in order to take up glucosides efficiently. Usually, IGs can be subjected to degradation by β-glucosidases in the plant tissue and insect gut producing reactive dialdehydes [47], whereas GSs are degraded by β-thioglucosidases (myrosinases) released in the plants after tissue disruption, which results in the formation of toxic volatile products [48]. The mechanism by which *Athalia* species avoid this degradation is only partially understood [49].

Next to glucoside sequestration of larvae, adults of *A. rosae ruficornis* are stimulated by clerodendrins and sequester these compounds from plants for mating and defense [50]. Here we show that adults of five European *Athalia* species were also stimulated by clerodendrin B, regardless of the larval host plant association (Table 3). Clerodane diterpenoids have not been described for Brassicaceae but occur in Lamiales [29], for example, in the genus *Scutellaria* (Lamiaceae), which also contains IGs [51]. Several *Athalia* species are known to feed as larvae on Lamiales [23], [24]. The use of *neo*-clerodane diterpenoids by adults may thus also reflect an ancestral association of *Athalia* with Lamiales. Further investigations on the role of *neo*-clerodane diterpenoids for larvae and adults of these *Athalia* species may reveal the complexity of utilizing several secondary metabolites for different ecological purposes.

Our molecular phylogeny based on about one third of known *Athalia* species revealed *A. scutellariae*, which feeds on Lamiales [23], [24], as the sister group of all remaining ingroup species used in this study (Fig. 2). Furthermore, the phylogeny indicates that multiple host shifts must have occurred. Both scenarios, a shift from the Lamiales to the Brassicaceae or the other way round, would require the same number of switches (Fig. 2). A more complete species sampling will be necessary to draw further conclusions on ancestral state relationships.

Several hypotheses have been put forward to explain host plant shifts in herbivorous insects, which fit well to the current findings in *Athalia*. According to the escape and radiation hypothesis, adaptation of insect species to novel plant chemical defenses should be followed by a burst of speciation [5], [52], [53]. The overall higher number (about 45) of *Athalia* species known to feed on Brassicaceae compared to only 14 species feeding on Lamiales [23], [54], [55] is concordant with the assumption that the association with Lamiales is ancestral and a radiation at the genus level occurred after switching to Brassicaceae. Thus, our data support the escape and radiation concept. Furthermore, our chemical analyses of *Athalia* adults preserved up to 15 years revealed several so far unknown host associations of various species with Brassicales and of only one species with Lamiales. Of course, we cannot exclude the possibility that these species also feed on other plant species. However, most *Athalia* species (except *A. circularis*, see Table 1) are described to be restricted to one plant order [23], [24]. Therefore, the chemical analysis of preserved adults, even by taking only one specimen per species, is an opportunity to reveal host plant relationships within this sawfly genus that shows, as far as is currently known, a high degree of host plant specialization.

In addition, the proposed host plant shift from Lamiales to Brassicaceae may be explained by the oscillation hypothesis [13], which proposes that derived herbivorous species may include plant species of several families for a certain period of time during their evolution. Larvae of *A. rosae rosae* fed at least somewhat on *P. lanceolata* and *V. beccabunga* leaves (Plantaginaceae) in no-choice tests, whereas *A. cordata* and *A. circularis* larvae never accepted the Brassicaceae *S. alba* or *B. rapa* [56]. Likewise, *A. rosae rosae* fed also comparably more on leaf discs treated with IG than *A. cordata* on discs treated with GS, although both clearly preferred the control discs (host plant treated with solvent only; Table 2). This finding indicates that *A. rosae rosae* has a higher host acceptance and that IGs are not entirely “novel” to this species, which falls in line with predictions of the oscillation hypothesis.

Apart from chemical similarities, geographical or ecological factors can influence host plant switches [57], [58]. One important prerequisite for a possible host plant switch is the sympatric distribution of herbivores and certain plant species [11], [59]. In our scenario in the sawfly genus *Athalia*, a shift, e.g., from *V. beccabunga* to *N. officinale* (Brassicaceae) might be conceivable, as these species share the same habitat in wetlands near rivers [60]. Similarly, in another herbivorous genus, the chrysomelid beetles *Phaedon armoraciae* L. and *Phaedon cochleariae* F., either one of these plant species serves as host [61]. Thus, the use of these two distantly related plant families may be more common among herbivorous insects. With regard to ecological factors, using specific secondary plant compounds for defensive purposes should reduce the likelihood of major host shifts [62]. However, the ability to gain new defense metabolites through sequestration from another plant species may help to reduce predation pressure to a similar extent [63], [64].

These hypotheses for host shifts in the genus *Athalia* are not mutually exclusive and may all have played a role in the evolution of these insect-plant associations. Overall, the host plant specificity of *Athalia* species is highly complex, and host plant chemistry has played a major role in the evolution of these herbivorous insects.

## Supporting Information

Table S1 Collection data for the sawfly specimens used in the molecular study and GenBank accession numbers.
(XLS)
